# Assessment of smoking status based on cotinine levels in nasal lavage fluid

**DOI:** 10.1186/1617-9625-5-11

**Published:** 2009-07-03

**Authors:** Mehmet Hakan Ozdener, Karen K Yee, Ryan McDermott, Beverly J Cowart, Aldona A Vainius, Pamela Dalton, Nancy E Rawson

**Affiliations:** 1 Monell Chemical Senses Center, Philadelphia, Pennsylvania, USA; 2 Dept Otolaryngology, Head-Neck Surgery, Thomas Jefferson University, Philadelphia, Pennsylvania, USA; 3 WellGen, Inc, North Brunswick, New Jersey, USA

## Abstract

Cotinine is a principal metabolite of nicotine with a substantially longer half-life, and cotinine levels in saliva, urine or serum are widely used to validate self-reported smoking status. The nasal cavity and olfactory system are directly exposed to tobacco smoke in smokers and in non-smokers who live with or work around smokers. However, despite the potential for a direct impact of tobacco smoke on the nasal epithelium and olfactory neurons, no prior studies have assessed cotinine levels in nasal mucus. We sought to determine whether cotinine levels in nasal lavage fluid (NLF) would provide a reasonable estimate of smoke exposure. We assayed cotinine using a competitive immunoassay in NLF from 23 smokers, 10 non-smokers exposed to tobacco smoke (ETS) and 60 non-smokers who did not report smoke exposure. NLF cotinine levels were significantly higher in smokers than in non-smokers, regardless of their exposure to ambient tobacco smoke. Cotinine levels in this small group of exposed non-smokers were not significantly different than those of non-exposed non-smokers. A cutoff of 1 ng/ml provided a sensitivity of 91% and a specificity of 99% for smoking status in this sample. Data were consistent with self-reported smoking status, and a cutoff of 1.0 ng/ml NLF cotinine may be used to classify smoking status. While saliva is the most easily obtained body fluid, NLF can be used to provide an objective and precise indication of smoking status and more directly reflects smoke exposure in the nasal and olfactory mucosa.

## Introduction

Precise estimation of direct exposure to tobacco smoke is a problem for epidemiologic studies due to human errors and inaccuracy in self report. Assessment of passive exposure to tobacco smoke is even more problematic [1,2]. While nicotine has a relatively short half-life of about 2 hours, cotinine, a principal metabolite of nicotine, has a half-life of approximately 20 hours, and is a specific and sensitive marker for determining exposure to tobacco [3-5]. Therefore, measurement of salivary, urinary or serum cotinine values have been used to validate self-reported smoking status [1,4], with saliva providing the most easily obtained source [6-8].

Notably, the olfactory sensory neuroepithelium and nasal mucosa are directly exposed to tobacco smoke in both smokers and non-smokers who live with or work around smokers. Smoking has been shown to reduce olfactory sensitivity in a dose- and time-dependent manner [9-12], and passive smoke exposure has been implicated in reduced olfactory function as well [13]. Using nicotine nasal spray caused adverse effects of nasal irritation and burning and taste and smell complaints [14]. Moreover, both exposures to tobacco smoke and to lipopolysaccharide, an active component of cigarette smoke, trigger a dramatic increase in the degree of olfactory neuron apoptosis [15,16].

The impact of smoking on the nasal mucosa has received considerably less study than its impact on lower respiratory tissue. Nonetheless, there is evidence for multiple deleterious effects, including increased nasal resistance, decreased mucociliary flow and mucosal sensitivity, and induces increase in DNA adduct and may cause nasal tumors due to numerous chemicals found [17,18]. Histopathological analysis of nasal mucosa obtained from rats exposed to tobacco smoke revealed a decrease in the extent of olfactory epithelium including loss of cilia and development of metaplasia [19].

Tobacco smoke may exert direct effects on nasal epithelial health and olfactory neuronal function, and proteomic analysis has demonstrated altered protein levels in the nasal lavage fluid (NLF) of smokers [20], yet no prior studies have assessed cotinine levels in nasal mucus. Basic information concerning cotinine levels in the NLF of individuals with varying degrees of exposure to tobacco smoke is of clinical importance for studies evaluating the impact of tobacco smoke on nasal and olfactory pathophysiology, as well as in other situations where a noninvasive sample is desired and saliva is not available or reliable. Therefore, we sought to determine whether cotinine levels in NLF would provide a reasonable estimate of smoke exposure. Our results indicate that NLF cotinine was significantly higher in smokers than in nonsmokers and established a cut of 1.0 ng/ml that may be used in future studies as an objective indicator of current smoking.

## Materials and methods

### Participants

Individuals (n = 97) who self-reported good general health participated. All were asked to complete a questionnaire regarding their smoking status (current smoker, past smoker, and non-smoker), smoking history (number of cigarettes or cigars smoked per day), smoking duration (years), if a past smoker, years since quitting, and ambient smoke exposure (type and duration). Four participants were excluded from the analyses: 2 provided inconsistent/incomplete information regarding their smoking habits, and 2 reported having recently quit smoking, and thus did not clearly fall into any of the smoking status groups described below. The remaining participants included 41 females and 52 males; their mean age was 36.1 ± 11.9 years (range 18–64 years).

### Questionnaire content

We used detail questionnaires to determine smoking status of subjects. Questions covered age, sex, smoking history, for smokers only, the number of cigarettes smoked per day, pipe or cigar smoking, number of years as a regular smoker. For non-smokers, when did you quit? How long did you smoke and what did you smoke? Exposure to environmental tobacco smoke was assessed by the following questions: "Who is currently a smoker, among people around you?"; "Do you live with someone who smokes? (Yes/No)"; and "In work place buildings, are you exposed to other people's tobacco smoke? (Yes/No).

### Smoking status groups

#### Smoker

Twenty-three subjects (8 females, 15 males) reported themselves to be current smokers. Active smokers consumed between 1 and 20 cigarettes/day at the time of assessment. Their mean age was 32.3 ± 9.1 years (range 21–64).

#### Non-smoker non-exposed

Sixtysubjects (27 females, 33 males) reported themselves to be non-smokers with a minimal history of ambient exposure to cigarette smoke. If prior smokers, the non-smokers had not smoked for at least 6 months and were not subject to substantial exposure to tobacco smoke in public or private surroundings. Their mean age was 37.9 ± 12.5 years (range 21–63).

#### Non-smoker exposed to tobacco smoke (ETS)

Ten passive smokers (6 females, 4 males) were defined by self-reported significant exposure to smoke in public buildings or private surroundings on a regular basis. Their mean age was 34.0 ± 12.2 years (range 18–53).

#### Collection of nasal lavage fluid

Subjects were given a sterilized metered-pump aerosolizer filled with 0.1 M sterile phosphate buffer solution without calcium or magnesium. Each pump action delivered 100 μl of solution (4 ml total per nostril). They were asked to spray and sniff 4–5 times into one nostril while occluding the other nostril, then to forcibly expel the nasal contents into a glass container. Collected NLF was then centrifuged at 9000 rpm for 10 minutes and the supernatant frozen at -20°C. Freezing NLF samples precipitates the mucins. On the day of the assay, samples were thawed completely, vortexed and centrifuged at 1500 × g (@3000 rpm) for 15 minutes. Samples were at room temperature before being added to the assay plate.

### Immunoassay of Cotinine concentration

To determine cotinine levels in NLF, High Sensitivity Salivary Cotinine Quantitative enzyme immunoassay kit (Cat. # 1–2112) from The Salimetrics™ was used according to manufacturer's instructions with slight modification. The test principle for this kit is based on the competition of cotinine for antibody binding sites. The cotinine in samples and peroxidase-labeled cotinine compete; therefore the amount of antibody bound to the plate is inversely proportional to the cotinine concentration.

Briefly, 20 μL of controls (blank and standards) and samples were pipetted into appropriate wells. First, enzyme conjugate and, second, antiserum were added and incubated for 1.5 hours at 37°C with constant mixing at 500–600 rpm. At the end of the incubation period, 3,3',5,5' Tetramethylbenzidine (TMB) solution was added and mixed at 500 rpm for 5 minutes and then incubated in the dark for an additional 25 minutes at room temperature. The reaction was quenched by adding stop solution using a multi-channel pipette, followed by mixing on a plate rotator at room temperature for 3 minutes at 500 rpm (or until the color turned from green to yellow). The activity of the peroxidase was determined by a distinct development of colors and detected by 450 nm extinction measurement with TMB substrate. The plate was read within 10 minutes of adding stop solution. These analyses took place at a room temperature between 20 and 25°C. Average optical density (OD) was calculated by subtracting the average OD for the non-specific binding wells from the average OD of the zero standards, controls, and unknowns. The percent bound (B/Bo) for each standard (200 ng/mL, 66.7 ng/mL, 22.2 ng/mL, 7.4 ng/mL, 2.5 ng/mL, and 0.8 ng/mL), control, and unknown was calculated by dividing the average OD (B) by the average OD for the zero (Bo). Final OD was converted to ng/ml.

### Statistical analysis

All analyses were performed using Systat^®^, version 11.00.01. Cotinine levels were not normally distributed (Shapiro-Wilk statistic = 0.54, p < 0.001). Therefore, pair wise comparisons between groups (smoking status and gender) were made using the non-parametric Kolmogorov-Smirnov Two Sample test. The Spearman correlation was used to assess the relationship between smoking frequency and NLF cotinine in smokers.

## Results

**Figure 1:**
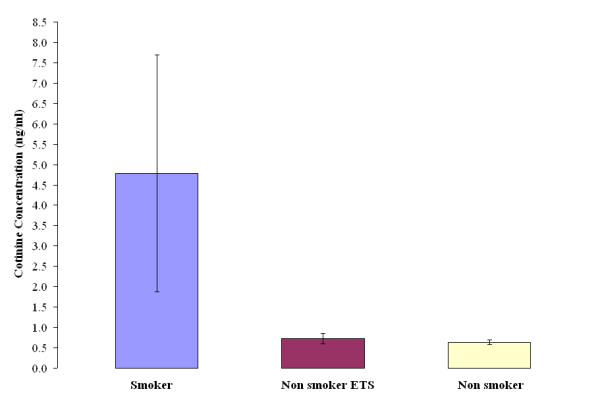
**Cotinine level is of indicator smoking status**. The median cotinine concentration in the NLF of non-smokers was 0.65 ng/ml, of passive smokers 0.64 ng/ml and of active smokers 4.78 ng/ml. Cotinine levels were significantly higher in the NLF of smokers than of that of either non-smokers non-exposed (p < 0.001) or non-smokers ETS (p < 0.001). No significant difference in cotinine levels between the non-smoker and nonsmoker ETS groups.

NLF cotinine levels ranged from 0.08 ng/ml to 11.73 ng/ml and averaged 7.5 times higher in smokers than in nonsmokers. The median cotinine concentration in the NLF of non-smokers was 0.65 ng/ml (0.08 ng/ml to 0.90 ng/ml), of passive smokers 0.64 ng/ml (0.54 ng/ml to 1.02 ng/ml) and of active smokers 4.78 ng/ml (0.90 n/ml to 11.73 ng/ml); the percentile distributions of levels in each group are given in Table 1. Cotinine levels were significantly higher in the NLF of smokers than of that of either non-smokers non-exposed (p < 0.001) or non-smokers ETS (p < 0.001) (Figure 1). There was, however, no significant difference in cotinine levels between the latter two groups. Although others have reported differences between non-smokers non-exposed and non-smokers ETS in cotinine levels in serum, saliva and urine [21-25], this is not a consistent finding, and there tends to be considerable overlap between these groups [5,24,25], probably reflecting, in part, difficulties in quantifying environmental smoke exposure.

**Table 1:** The percentile distributions of cotinine levels in NLF

**Self-reported smoking habit**	**N**	**Min**	**10th**	**25th**	**50th**	**75th**	**90**^**th**^	**Max**
**Nonsmokers**	60	0.00	0.56	0.59	0.65	0.71	0.76	0.90

**Nonsmokers ETS**	10	0.54	0.59	0.60	0.64	0.86	0.98	1.02

**Smokers**	23	0.9	1.11	1.70	4.27	7.51	10.41	11.73

Among non-smoking non-exposed subjects, there was a small but significant difference between females and males, with females showing slightly lower cotinine levels, as has been reported by others [23,26]. However, no significant gender difference was observed in our smaller groups of passive or active smokers, nor was there a gender difference among smokers in reported frequency of smoking.

**Figure 2:**
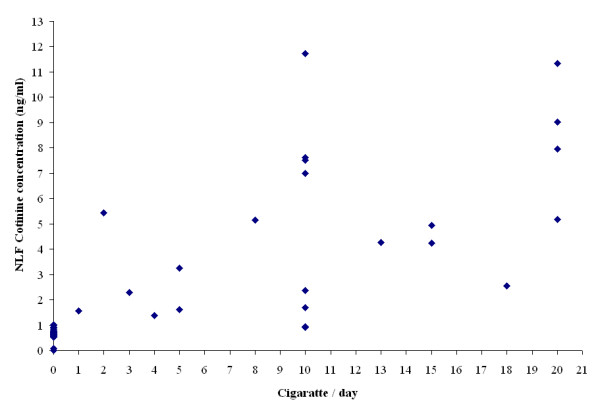
**Association between the concentration of NLF cotinine and the number of cigarettes reported to be smoked daily**. Significant correlation was found between reported frequency of cigarette use and measured NLF cotinine levels in smokers.

There was a significant correlation (rho = 0.48, p < 0.02) between reported frequency of cigarette use and measured NLF cotinine levels in smokers (Figure 2). This is consistent with reports of cotinine levels in saliva and serum [27,28]. Of note, cotinine levels observed in the two participants excluded from the general analyses who reported recent abstinence from smoking (<3 months) were consistent with, or higher than, their reports of prior usage (1 cigarette/week, cotinine = 1.54 ng/ml; 20 cigarettes/day, cotinine = 8.37 mg/ml), suggesting either unreported use of nicotine containing products, misreport of current usage or slow clearance of cotinine in the nasal mucous.

**Figure 3:**
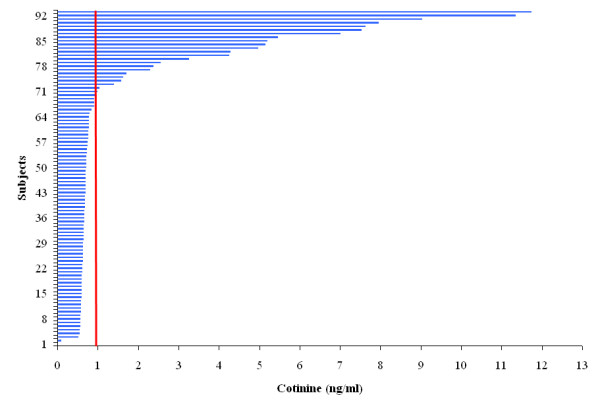
**Distribution of NLF cotinine across all subjects**. NLF cotinine level of 1 ng/mL was lowest concentration as a cutoff point to distinguish nonsmokers from smokers with the best combined levels of sensitivity (91%) and specificity (99%). Similar cutoff levels were obtained in men and women (not shown). NLF cotinine levels lower than 1 ng/mL were observed in 100% of the self-reported non-exposed nonsmokers and in more than 90% of self-reported nonsmokers ETS.

Based on self-reported smoking status, we estimated the lowest concentration of cotinine as a cutoff point to distinguish nonsmokers from smokers. The distributions of NLF cotinine in smokers and nonsmokers (exposed and not exposed) overlapped in few subjects. The value of 1 ng/mL represented the best combined levels of sensitivity (91%) and specificity (99%) (Figure 3). The sum of sensitivity and specificity changed little for cutoff values between 1 ng/ml (sensitivity, 91%; specificity, 99%) and 2 ng/ml (sensitivity, 74%; specificity, 100%). Similar cutoff levels were obtained in men and women (not shown). NLF cotinine levels lower than 1 ng/mL were observed in 100% of the self-reported non-exposed nonsmokers and in more than 90% of self-reported nonsmokers ETS. In smokers, fewer than 10 percent of smokers had levels lower than 1 ng./ml.

## Discussion

The effects of tobacco smoke on the xenobiotic enzymes (cytochrome P-450 system) in the olfactory tissues are of particular interest because nasal tissues, in particular, olfactory epithelium, are an initial contact site with inhaled toxicants. Nicotine is rapidly metabolized to cotinine via the cytochrome P450 pathway, which is active within cells in both the nasal and olfactory epithelia. In humans nicotine is mainly converted to cotinine by cytochrome P450 (CYP2A) in human olfactory epithelium [29]. Previous studies demonstrated that cotinine levels may vary in body fluids in relation to differences in exposure and the activity of xenobiotic metabolizing enzymes in different tissues and organs [10,15]. The time course of nicotine in the body organs and resultant pharmacologic effects are highly dependent on the route and rate of dosing. Nicotine accumulation is age-dependent and is quantitatively different in various segments in the mouse brain [30]. Nicotine also accumulates markedly in different body fluids such gastric fluid, saliva and breast milk. Comparison of these gastric fluid/plasma, saliva/plasma and milk/plasma ratio was found different depend on administration route [31,32]. Additionally, changes in the expression of nasal xenobiotic metabolizing enzymes due to tobacco smoke may have a role in the development of deficits in olfactory performance. Interestingly, xenobiotic-metabolizing enzymines of the nasal mucosae may be regulated differently than other tissues [33].

Thus, differences in the levels of cotinine in the nasal mucus may reflect both exposure to nicotine and the ability of the nasal detoxification system to metabolize this xenobiotic chemical. Two major biomarkers for assessment of smoking status, nicotine and cotinine can be measured in various biological samples, and can be assayed using gas or liquid chromatography, radioimmunoassay (RIA) or enzyme immunoassays (ELISA) [34-37]. However, there has been no report of the whether cotinine can be assayed in NLF, and if so, whether levels reflect smoking status of the subject with sufficient accuracy for use in confirmation of smoking status. Our data provide a reliable protocol and reference criteria for assignment of smoking status based on cotinine levels in nasal lavage fluid. In conclusion, NLF cotinine can be a reliable biomarker of clinical importance in studies evaluating the impact of nicotine exposure on nasal and olfactory pathophysiology, as well as in other situations where saliva is not available and an objective indicator of smoking status is needed.

## Abbreviations

NLF: Nasal lavage fluid; ETS: Exposed totobacco smoke.

## Competing interests

The authors declare that they have no competing interests.

## Authors' contributions

MHO carried out most of experiments and drafted the manuscript. KKY, RM and AAV participated in the design of the study and collected samples. PD and BJC helped to organize and analyze the data and edit the manuscript. NER initiated and managed the project and coordinated and edited the manuscript. All authors read and approved the final manuscript.
